# Altered PTEN expression; a diagnostic marker for differentiating normal, hyperplastic and neoplastic endometrium

**DOI:** 10.1186/1746-1596-4-41

**Published:** 2009-11-25

**Authors:** Soheila Sarmadi, Narges Izadi-Mood, Kambiz Sotoudeh, Seyed Mohammad Tavangar

**Affiliations:** 1 Department of Pathology, Mirza Koochak Khan Hospital, Tehran University of Medical Sciences, Tehran, Iran; 2 Resident of Pathology, Iran University of Medical Sciences, Tehran, Iran; 3 Department of Pathology, Shariati Hospital, Tehran University of Medical Sciences, Tehran, Iran

## Abstract

**Background:**

Different molecular alterations have been described in endometrioid endometrial carcinoma (EECA). Among them the most frequently altered is loss of the PTEN protein, a tumor suppressor gene. The purpose of this study was to evaluate the expression pattern of PTEN gene in normal, hyperplastic and neoplastic endometrium.

**Methods:**

In a study in a referral gynecologic hospital in Tehran, Iran, immunohistochemical (IHC) evaluation of PTEN was performed on 87 consecutive specimens to the following three groups; group A- normal proliferative endometrium(n = 29); group B- hyperplastic endometrium [including simple hyperplasia without atypia(n = 21) and complex hyperplasia with atypia (n = 8)] and group C- EECA(n = 29). Immunostaining of cells was analyzed by arbitrary quantitative methods according to both slide's area staining and intensity of color reaction.

**Results:**

PTEN immunoreactivity was present in all normal proliferative endometrium, all simple hyperplasia, 75% of atypical complex hyperplasia and in 48% of EECA (P < 0.001). The intensity of PTEN reaction was significantly higher in group with proliferative endometrium than hyperplastic endometrium and EECA (P < 0.001).

**Conclusion:**

PTEN expression was significantly higher in cyclical endometrium than in atypical hyperplasia and endometrioid carcinoma.

## Background

Endometrioid endometrial carcinoma (EECA) accounts for three fourths of endometrial cancers and are thought to develop following a continuum of premalignant lesions ranging from endometrial hyperplasia without atypia, to hyperplasia with atypia and finally to well differentiated carcinoma[1,2]. Based on light microscopic appearance and clinical behavior, endometrial cancers have long been classified into major categories (type I and II) [2-4]. Accurate diagnosis of premalignant lesions in routine endometrial biopsies has a great clinical value in patient management. Unfortunately several recent studies have shown that cytological atypia which is predominant criterion for diagnosis of premalignant lesions (atypical endometrial hyperplasia), have poor reproducibility [1,3]. Therefore, solving these problems needs new insights into the morphology of biologically defined premalignant lesion of endometrium [1]. Recent molecular diagnostic methods have provided new ancillary tools for premalignant lesion diagnosis. EECA has a variety of genetic alternations, including microsatellite instability (MI) and mutations of PTEN, k-ras, and β-catenin genes [5,6]. Also, these molecular genetic alternations have been described in atypical endometrial hyperplasia [5]. Currently, PTEN is the most frequently altered gene in EECA which is located on chromosome 10 [6,5]. The PTEN gene has both lipid and protein phosphate activity and the combination of the losses of PTEN lipid and protein phosphate activity can cause an aberrant cell growth and an escape from apoptosis, as well as abnormal cell spreading and migration [6]. Up to 50% of all EECA and 83% of tumors with adjacent premalignant lesions show altered PTEN, characterized by loss of expression [5-7]. Mutations of PTEN are frequently detected in several cancers such as: endometrium [8-13], low grade endometrioid ovarian carcinoma (20%) that is the second most common histological subtype of ovarian cancer [14] prostate [15], breast [16], and glial tumors [5,17,18]. Among the different histological subtypes of EECA, endometrial subtypes have the highest frequency (34-80%) of PTEN mutations [19]. PTEN - null glands (i.e., loss of PTEN expression) are shown in a diffuse pattern in EECA but also may be detected in morphologically normal endometrial tissue, which suggests that PTEN alternation occur in the earliest phase of endometrial carcinogenesis [5,17,20]. Immunohistochemical detection of PTEN in cycling endometrium reveals high levels of protein expression in all different cell types during the proliferative phase, with diminution or absence of PTEN protein expression in mid secretory glands [1,17,21,22]. The hypothesis that loss of PTEN expression could be assessed by immunohistochemical method has led to the suggestion that PTEN immunostaining may be a new and effective tool for screening of malignant and premalignant endometrial lesions [11,23].

In the present study we used immunohistochemical method to evaluate PTEN expression in three groups of specimens from normal, hyperplastic endometrium and EECA.

## Methods

Ninety paraffin-embedded endometrial tissue samples diagnosed as: normal proliferative endometrium consisting of 14 and 16 early and late proliferative endometrium, respectively, endometrial hyperplasia including: 22 simple hyperplasia (SH) and 8 atypical complex hyperplasia (ACH) and 30 EECA were selected from surgical pathology files of the department of pathology of Mirza Koochak Khan Hospital, a referral gynecological hospital in Tehran, Iran. Prior to data collection, the study was reviewed and approved by the university ethical committee. All the selected samples in the present study were curettage specimens. Hematoxylin-eosin-stained sections from each case were reviewed by an expert pathologist (NIM) to confirm the histological diagnosis. Hyperplasic specimens were evaluated according to the WHO histological classification, (WHO 94) [24]. Specimens with any evidence of chronic nonspecific endometritis, endometrial polyp, secretory changes or progesterone effect were excluded and the most representative paraffin block for each case was then selected for immunohistochemical analysis.

Immunohistochemical study: Sections of 4 μm in thickness were deparaffinized in xylene and rehydrated through a series of graded alcohols. Antigen retrieval was achieved by heat treatment at 98 centigrade's in PT module buffer 1 (citrate buffer, PH = 6.0) for 20 minutes. Endogenous peroxidase activity was blocked by incubating slides in serum blocking solution. The sections were incubated with anti-PTEN polyclonal antibody (Zymed Laboratories, South San Francisco, CA, USA) and diluted 1: 100 in phosphate buffer, for 60 minutes respectively and then incubated in enzyme conjugate for 10 minutes. The reaction was visualized with the Zymed immunohistochemical detection kit using diaminobenzidine chromogene as substrate. Finally, the sections were counterstained with Mayer's hematoxylin. Normal prostate tissue was used as positive control and the negative control was performed without addition of the primary antibody. Immunohistochemical slides were evaluated synchronously by two pathologists under light microscope and uniform criteria were used. Immunoreactivity was regarded as positive when brown staining was localized in the nuclei or cytoplasm of normal endometrial glandular cell (an internal positive control) or tumoral cell. According to Kapucuoglu et al [5] and An et al [8] the immunoreactivity was graded arbitrarily and semi quantitatively by considering the percentage and intensity of staining on the whole section. Staining of cells was scored as negative if < 10%, + 1 if 10%-50% and +2 if >50% of slide's area was stained positive [25]. The intensity of PTEN staining was scored from 0 = absent, +1 = light brown, +2 = brown to dark brown in the nucleous or cytoplasm of glandular cells for each specimen.

Statistical analyses were carried out by Statistical Package for Social Sciences v 15.0 (SPSS Inc., Chicago, IL, USA) software for Windows using Chi-square, ANOVA and Kruscal-Wallis tests. P value < 0.05 was considered significant.

## Results

From 90 cases of the immunohistochemistry staining, 3 cases (1 case from each group: early proliferative, SH and EECA) were excluded because there were no representative tissue samples after immunohistochemistry staining.

PTEN immunoreactivity was noted in all normal proliferative endometrium (29/29,100%) and SH (21/21,100%). In ACH and EECA immunoreactivity was positive in 6 (6/8, 75%) and 14 (14/29, 48%) cases respectively (Tables 1 and 2). The difference of immunoreactivity between the groups were significant (P < 0.001).

**Table 1:** PTEN expression based on the slide's area staining

Type of endometrium	Slide Area		
	< 10%	10-50%	>50%
***Proliferative Endometrium (n)***			
Early	0	0	13
Late	0	0	16
***Hyperplasia (n)***			
Simple	0	4	17
Atypical Complex	2	3	3
***Carcinoma (n)***			
EECA	15	3	11

**Table 2:** PTEN expression based on the intensity of color reaction

Type of endometrium	Color Intensity*		
	0	+1	+2
***Proliferative Endometrium (n)***			
Early	0	1	12
Late	0	2	14
***Hyperplasia (n)***			
Simple	0	7	14
Atypical Complex	2	3	3
***Carcinoma (n)***			
EECA	15	8	6

*0 = absent, **+1 **= light brown, **+2 **= brown to dark brown

**Figure 1:**
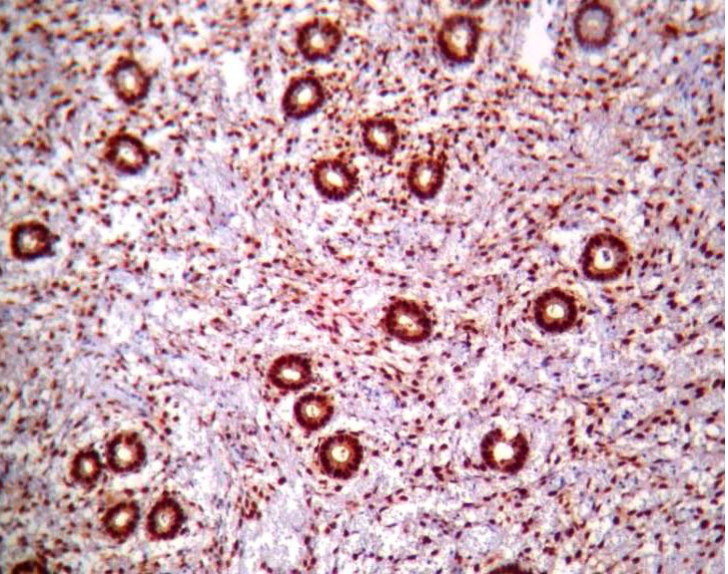
**Immunohistochemical staining using PTEN antibody showing strong and diffuse positivity in normal proliferative endometrium, (PTEN, ×100)**.

**Figure 2:**
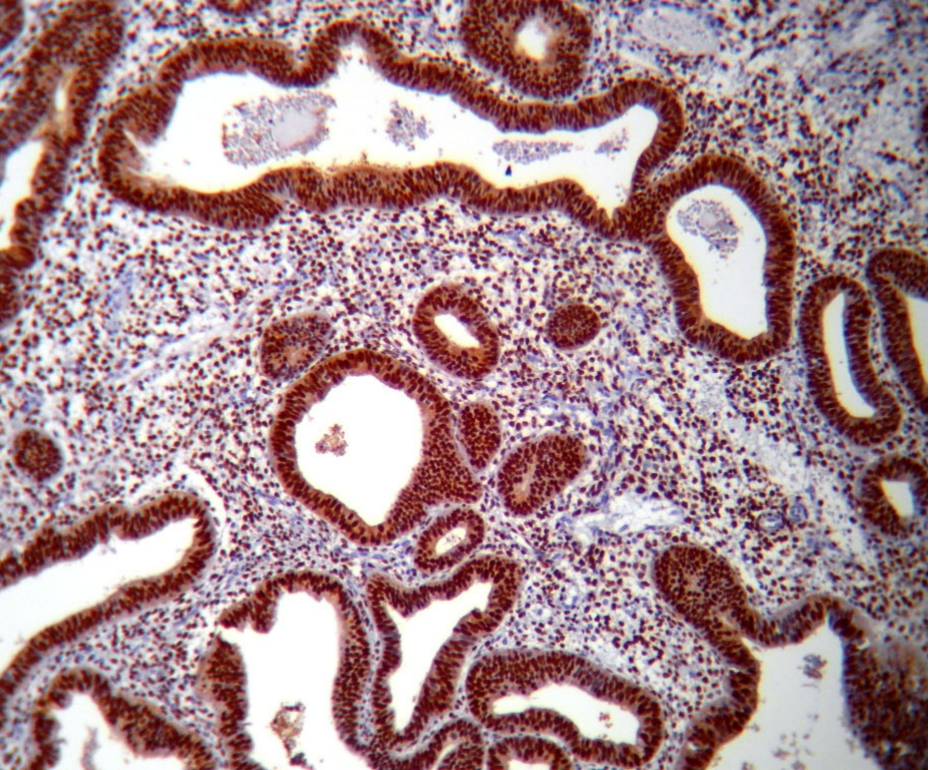
**Immunohistochemical staining using PTEN antibody showing strong reactivity of simple hyperplasia of endometrium, (PTEN, ×100)**.

**Figure 3:**
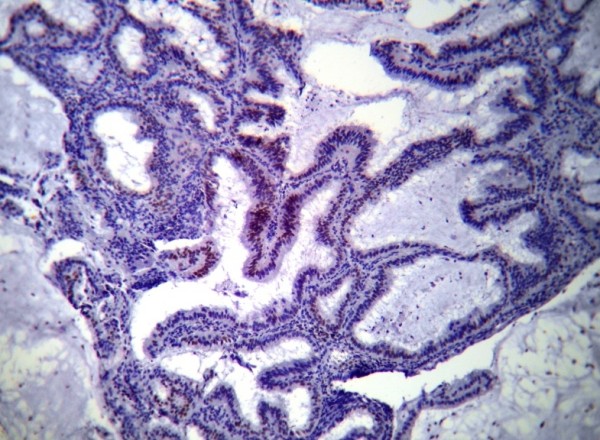
**Immunohistochemical staining using PTEN antibody showing weak and focal reaction in atypical complex hyperplasia of endometrium, (PTEN, ×100)**.

**Figure 4:**
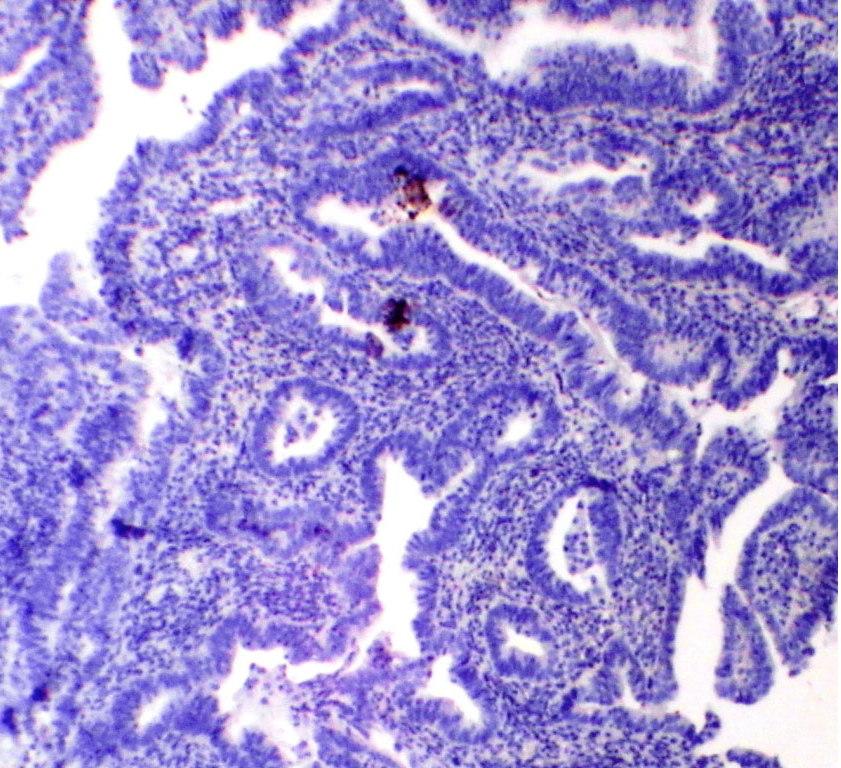
**Immunohistochemical staining using PTEN antibody showing complete lack of reaction in endometrioid endometrial carcinoma, (PTEN, ×100)**.

PTEN immunoreactivity was heterogeneous. Some cells within a gland or some glands were negative for PTEN staining respectively in ACH & EECA. The normal proliferative endometrium showed intense cytoplasm and/or nucleus staining in the glandular epithelial cells (Figure 1). The lowest PTEN immunoreactivity was detected in EECA and the differences were significant (P < 0.001). The PTEN expression in cyclical endometrium and SH (Figure 2) was higher than in ACH and EECA (Figure 3, 4). There was no statistically significant difference between the PTEN expression in early and late proliferative endometrium, SH and also between ACH and EECA.

## Discussion

Endometrial carcinoma is the fifth most common cancer of women worldwide [19].

Based on clinicophatologic observations, there are two types of endometrial carcinoma: Type-I usually arising in the background of endometrial hyperplasia and Type - 2 which is unrelated to estrogen [2].

Endometrial hyperplasia is classified by the WHO into four groups, namely simple hyperplasia, simple hyperplasia with atypia, complex hyperplasia, and complex hyperplasia with atypia. Currently, there is a lack of criteria that could accurately predict the disease outcome and there is need for a new classification composed of three groups: endometrial hyperplasia (EH), endometrial intraepithelial neoplasm (EIN) and endometrial carcinoma [18,26,27]. The pathogenesis of endometrial carcinoma and its precursor lesion is complex and involves many molecular disturbances. The most frequently altered gene in endometrial tumors of endometrioid histology showing microsatellite instability is PTEN inactivation and several studies have found that PTEN inactivation is correlated with clonal growth patterns detected in endometrial hyperplasia and carcinoma [25,28,29]. In the present study, PTEN negative immunoreactivity was detected in the majority of EECA and ACH but none in typical SH and normal proliferative endometrium.

Mutter et al examined the altered PTEN expression in endometrial tissue samples.

PTEN expression in 61% (20 of 33) of cases was completely absent and 97% (32 of 33) of cases revealed at least some diminution in expression [11]. Allison et al showed that biomarkers alone or in combination had most consistency to make a clear distinction between normal, endometrial hyperplasia and EECA. They have concluded that the combination of loss of PTEN expression and particular histological features have got the greatest diagnostic utility in endometrial hyperplasia [30].

In the present study, we detected loss of PTEN expression in 52% of EECA and 25% of ACH. Orbo et al, [12] reported loss of PTEN protein expression in 55% of specimens in patients with subsequent EECA and Kapucuoglu et al [5] found complete loss of PTEN in 20% of atypical complex samples. The results of present study are comparable to reported studies.

Except for 2 totally PTEN- negative cases of ACH, PTEN immunoreactivity was heterogeneous in most of our patients with ACH, in which PTEN- negative hyperplastic glands were scattered among PTEN- positive glands.

This intermittent pattern was detected at a variety of glands densities, ranging from the null gland with closely packed architecture to PTEN- positive glands in the low densities of a disordered proliferative endometrium. The cytology of PTEN- nonexpressing glands was atypical which different from the disordered glands. We found a less frequent pattern of heterogeneous PTEN staining in some benign precancer samples without cytological changes. Based on previous studies and our experience, it is obvious that loss of PTEN expression begins in the earliest stages of endometrial tumorigenesis, under conditions of excesses estrogen exposure [11]. PTEN inactivation initiate in precancers from a normal background state, and additional PTEN damage accumulates in the transition from premalignant to malignant disease.

In our study PTEN positivity was found in all proliferative endometrium with no differences between early and late proliferative phases, and the highest PTEN immunoreactivity as well as homogeneity were detected in normal proliferative endometrium. Also all cases of endometrial hyperplasia without atypia were PTEN positive with high immunoreactivity, but in contrast to normal proliferative had less homogenous pattern, in which a few scattered PTEN negative hyperplastic glands are interposed among PTEN positive hyperplastic glands.

We found significant differences in PTEN expression between proliferative endometrium, EECA and also between SH and ACH.

Based on the histopathologic criteria differentiation between EECA and ACH may be very difficult and the reproducibility of the WHO classification in the diagnosis of hyperplasia is disappointing [5,31].

Our results are comparable to mentioned studies, because we found no statistically significant differences between PTEN expressions in ACH and EECA.

Immunohistochemical identification of individual PTEN-null glands in endometrium with excesses estrogen exposure may be help to detection of precancers to an earliest stage of malignancy. In regard to hyperplasia and atypia, our results showed lower PTEN activity (25%) than in other studies (55-75%); which may be due to use of polyclonal antibody in our study. As Pallares et al showed, using monoclonal antibody was associated with more acceptable results than polyclonal antibody [23].

In conclusion, decreased PTEN expression tended to associate with malignant features of endometrium with significant statistical difference of PTEN immunoreactivity between groups of normal endometrium, hyperplastic changes & carcinoma. Our data suggested that loss of PTEN expression is partly associated with the endometrial cancers through a premalignant phase.

## Competing interests

The authors declare that they have no competing interests.

## Authors' contributions

SS designed the study and participated in histological diagnosis, writing and revising the manuscript and performed immunohistochemistry analysis and interpretation. NIM participated in histological diagnosis, performed immunohistochemistry analysis and interpretation. KS participated in writing and editing of manuscript, performed data analysis and interpretation of statistical data. SMT carried out immunohistochemistry. All authors read and approved the final manuscript.
